# Progress in the treatment of lung adenocarcinoma by integrated traditional Chinese and Western medicine

**DOI:** 10.3389/fmed.2023.1323344

**Published:** 2024-01-08

**Authors:** Hongxin Jiang, Lina Bu

**Affiliations:** ^1^The College of Life Sciences, Northwest University, Xi’an, Shaanxi, China; ^2^Department of Respiratory and Critical Care Medicine, Xi’an No.3 Hospital, The Affiliated Hospital of Northwest University, Xi’an, China

**Keywords:** combined Chinese and Western medicine treatment, lung adenocarcinoma, review, Western medicine, Chinese medicine

## Abstract

Non-small cell lung cancer (NSCLC) overwhelmingly represents the predominant histological subtype of lung cancer, with lung adenocarcinoma emerging as the most prevalent form. Conventional Western medical treatments encompass a spectrum of modalities, including surgical interventions, cytotoxic chemotherapy, radiotherapy, targeted pharmacotherapy, and immunotherapy. In contrast, Traditional Chinese Medicine (TCM) methodologies encompass traditional Chinese medicine treatments, acupuncture therapies, and tuina treatments. While conventional Western medicine has made remarkable strides in the treatment of lung cancer, it is important to acknowledge the limitations inherent in singular treatment approaches. Consequently, the quest for a more comprehensive and integrative therapeutic paradigm becomes imperative. A deficiency of evaluation criteria specific to lung adenocarcinoma treatment in the realm of TCM represents an outstanding challenge in need of resolution. Nonetheless, in the backdrop of the continuous evolution of lung adenocarcinoma treatment modalities, the amalgamation of Chinese and Western medical approaches for treating this condition has exhibited a promising trajectory. It not only contributes to mitigating toxicity and augmenting efficacy but also serves to reduce a spectrum of postoperative complications, thereby enhancing the quality of patients’ survival and extending life expectancy. This article furnishes a comprehensive survey of the research advancements in the integration of Chinese and Western medical approaches for treating lung adenocarcinoma. It elucidates the merits and demerits of individual and combined therapeutic strategies, surmounts current limitations, underscores the virtues of amalgamating Chinese and Western medical paradigms, and offers a more holistic, integrated, and efficacious treatment blueprint.

## 1 Causes of lung adenocarcinoma

Lung cancer stands as the predominant malignant ailment in China, with a staggering annual incidence of 789,000 new cases and approximately 631 deaths (1). Non-small cell lung cancer, chiefly represented by lung adenocarcinoma, encompasses a spectrum of clinicopathological types, including invasive adenocarcinoma (IAC), invasive adenocarcinoma variants, micro-invasive adenocarcinoma (MIA), and pre-infiltrative lesions; the latter category further bifurcates into adenocarcinoma *in situ* (AIS) and Atypical adenoma hyperplasia (AAH) (2). Notably, early-stage lung adenocarcinoma patients typically remain asymptomatic and are often incidentally diagnosed during routine physical examinations. However, the highly infiltrative and metastatic nature of lung adenocarcinoma occasionally results in distant metastases upon diagnosis. Patients in the intermediate and advanced stages of the disease frequently manifest conspicuous symptoms, primarily characterized by hemoptysis, chest tightness, dyspnea, and bone pain, among others (2).The therapeutic management of lung adenocarcinoma in Western medicine varies based on disease staging. In the early stages, surgical resection assumes a pivotal role, achieving a curative effect through tumor excision. In contrast, late-stage patients typically undergo conventional chemotherapy, radiotherapy, targeted pharmacotherapy, and immunomodulation to gain control over the disease, alleviate symptoms, and extend survival (3).

Chinese medicine’s approach to lung adenocarcinoma centers on the modification and regulation of the body’s internal milieu using traditional Chinese medicine components. This approach aims to bolster immunity, reduce adverse reactions, and enhance clinical efficacy. Modalities include traditional Chinese medicine treatments, acupuncture, and tuina therapy, among others (4). While Western medicine has made substantial strides in treating lung adenocarcinoma in recent years, improving patient survival rates, the associated adverse reactions and toxic side effects detrimentally impact patients’ quality of life (5).

In contrast, Traditional Chinese Medicine (TCM) boasts several advantages in the treatment of lung adenocarcinoma. It emphasizes evidence-based and individualized treatment plans tailored to patients’ specific conditions (6). However, standardized diagnostic and therapeutic criteria for TCM treatment of lung adenocarcinoma have not been universally established, representing an ongoing challenge in this field (7).

To address the need for a more comprehensive and integrated approach, the combination of Chinese and Western medicine has emerged as a compelling research focus (8). This integrated approach capitalizes on Western medicine’s direct targeting of tumors and Chinese medicine’s role in enhancing immune function, thereby improving treatment compliance and efficacy. This synergistic approach not only reduces toxicity and enhances efficiency but also mitigates post-surgery complications, enhances the quality of life, and extends survival (9). The combination of Traditional Chinese Medicine and Western medicine in the management of lung adenocarcinoma represents a holistic and synergistic treatment strategy, harnessing the strengths of both paradigms. It takes into account patients’ specific conditions and physical status, facilitating the formulation of scientifically grounded treatment plans (10). Consequently, the combination of Chinese and Western medicine emerges as a superior approach to lung cancer treatment. This article offers a comprehensive review of the utilization of Chinese medicine, and Western medicine, and their synergistic combination in the recent treatment of lung adenocarcinoma.

### 1.1 Western medicine to the etiology of lung adenocarcinoma

Within the purview of Western medicine, the precise etiology of lung adenocarcinoma remains enigmatic, and its pathogenesis is postulated to stem from a myriad of factors, as gleaned from clinical observations (11). These contributory factors encompass an array of determinants, such as long-term tobacco smoking, environmental pollution, occupational exposures, genetic predisposition, and concomitant chronic respiratory ailments (12). Notably, while chronic tobacco smoke exposure ranks as the most prevalent cause, lung adenocarcinoma manifests in approximately 15–20% of non-smokers, often attributed to a complex interplay of environmental and genetic influences (9). Literature within the field has reported that variances in the germline ancestry of lung adenocarcinoma may also exert an influence on the subsequent selection of somatic mutations, thereby contributing to the development of diverse subtypes of this cancer (13).

Furthermore, obesity, recognized as a risk factor for various malignancies, has been posited to exert differential effects on the idiosyncratic incidence patterns among lung adenocarcinoma patients (14). Clinical presentations among individuals afflicted by lung adenocarcinoma predominantly encompass persistent cough, chest pain, hemoptysis, dyspnea, chest constriction, and fever.

### 1.2 Understanding of etiology and pathogenesis of lung adenocarcinoma in traditional Chinese medicine

From a TCM perspective, lung adenocarcinoma is perceived as a manifestation of the body’s internal imbalance, reflecting disturbances not only within the body but also in the intricate interplay with the external environment. This pathological state is characterized by the depletion of vital qi, internal deficiencies, external pathogenic factors, and a profound disruption in the harmonious equilibrium between yin and yang.

Under these conditions, malignancy can take root as malevolent influences exploit the body’s weakened state, infiltrating the lungs and precipitating dysfunctional pulmonary processes (15). Consequently, the disease primarily emerges as a consequence of deficiency and is considered a “real” ailment. The underlying root of this ailment lies in the deficiency of vital qi, with associated symptoms marked by the accumulation of phlegm, stagnation of qi, blood stasis, and the accumulation of toxic elements within the pulmonary system, all contributing to the pathogenesis of lung adenocarcinoma (16).

By the principles of Chinese medicine, the lung is associated with the emotion of sadness and is intrinsically linked to the water element, the source of phlegm. Women, often more susceptible to emotional distress and prone to stagnation, may experience deficiencies in vital qi, ultimately fostering the formation of pulmonary “rocks.” Notably, lung adenocarcinoma is typified by coldness, fluid coagulation, and a propensity toward qi stagnation and blood stasis, rendering it prone to metastasis. These factors collectively contribute to the higher incidence of lung adenocarcinoma among the female population (17).

## 2 Overview of lung adenocarcinoma treated by Western medicine

Lung adenocarcinoma, originating from lung epithelial cells, is generally less aggressive than small-cell lung cancer. Its development is typically gradual, often remaining asymptomatic in its early stages (18). The international TNM staging system classifies lung adenocarcinoma into four stages: stage I for early-stage disease and stages II, III, and IV for intermediate and advanced stages (19). Each stage and subtype of lung adenocarcinoma warrants specific treatment approaches.

### 2.1 Surgical treatment

In the early stages, radical surgical resection stands as the optimal treatment approach for tumors. For stage I lung adenocarcinoma with high-risk factors, the gold standard of treatment is lobectomy combined with mediastinal lymph node dissection (20). Older patients typically undergo wedge resection or lobectomy as the standard treatment. In cases of local and regional advancement, combining surgical intervention with drug therapy and radiotherapy has shown promise in enhancing patient survival (21). Surgical treatment offers the advantage of directly excising tumors and affected organs comprehensively, irrespective of cell proliferative status or treatment sensitivity. This approach aims to completely remove the tumor and associated local tissues while preserving healthy lung tissue, it can also better understand the situation of regional lymph node metastasis and effectively control the further enlargement and spread of lung cancer, thereby achieving a radical therapeutic outcome and enhancing the patient’s quality of survival.

### 2.2 Chemotherapy

Early-stage lung adenocarcinoma often presents with subtle or no clinical symptoms, and symptoms typically become evident in the middle or advanced stages of the disease. This necessitates systemic treatments, which can limit the applicability of surgical methods. Depending on disease type and stage, appropriate radiotherapy methods can be selected, and conventional chemotherapy is employed in various contexts, including neoadjuvant chemotherapy, postoperative chemotherapy, and combined radiotherapy and chemotherapy (22). Research has demonstrated that platinum-based adjuvant chemotherapy significantly enhances the survival rates of patients with completely resected stage II or stage III lung adenocarcinoma, particularly when tailored to individual patients through biomarker screening (23). Previous experiments have indicated that for stage IV lung adenocarcinoma patients, a radiotherapy regimen followed by a combination of cisplatin and other chemotherapy agents can enhance the efficacy of radiotherapy while mitigating its side effects (24). Postoperative chemotherapy plays a pivotal role in reducing the incidence of lung adenocarcinoma and extending patient survival. Systemic chemotherapy not only controls tumor growth, reduces symptoms, and improves quality of life, but also delays metastasis to other organs. Paclitaxel as a natural compound is widely used as a chemotherapeutic drug, by inhibiting mitosis and triggering apoptosis of cancer cells, thus effectively preventing the proliferation of cancer cells, with good anti-tumor effects and few side effects (25). Camptothecin is a quinoline alkaloid, as a natural antitumor drug, mainly sensitive to proliferating cells, blocking cell division of cell cycle-specific antitumor drugs, in combination with cisplatin can enhance the sensitivity of lung cancer cells to cisplatin (26).

### 2.3 Targeted drug therapy

Targeted drugs represent a potent therapeutic avenue for lung adenocarcinoma, precisely honing in on specific molecules within the cancer cells, including EGFR, ALK, KRAS, ROS1, BRAF, and more. This precision inhibits the growth and dissemination of cancerous cells effectively (27). However, drug resistance remains a challenge in the targeted treatment of lung adenocarcinoma, necessitating the exploration of new mutant genes to catalyze breakthroughs in this therapeutic approach (28). Emerging studies have identified three genes, namely CDCA8, MCM6, and TTK, as potential novel drug targets, harboring pivotal roles in restraining lung cancer cell proliferation, exerting anti-tumor activities, and potentially enhancing patient survival (29). Targeted drug therapy is a purposive treatment with the advantages of less damage to normal cells, less harm to the body, and better therapeutic effects.

### 2.4 Radiotherapy

Radiotherapy stands as an effective approach for alleviating symptoms and bolstering local lesion control in patients grappling with advanced lung adenocarcinoma. It achieves this by employing high-energy rays to eliminate tumor cells, thereby restraining tumor growth (30). Research has highlighted that, alongside induction systemic therapy and maintenance therapy, stereotactic radiotherapy substantially extends progression-free survival from 3.5 months to an impressive 9.7 months in lung adenocarcinoma patients (31). Additionally, the application of stereotactic radiotherapy in patients with low metastatic burden significantly enhances survival rates. This reflects not only a breakthrough in augmenting the survival prospects of advanced lung adenocarcinoma patients but also demonstrates the benefits of local consolidation interventions (32).

Previous investigations have demonstrated that employing moderate radiation therapy doses in lung adenocarcinoma patients can enhance overall survival without elevating the incidence of radiation pneumonitis (33). Radiation therapy is uniquely suited to treating multiple lesions throughout the body and having a low impact on the entire body due to its lack of site restriction as well as its lesser vascular restriction.

### 2.5 Immunotherapy

Amid the ongoing progress in molecular and immune research along with the development of related drugs, immunotherapy has swiftly emerged as a linchpin in the management of operable and metastatic lung adenocarcinoma. This represents a pivotal advancement in lung adenocarcinoma treatment in recent years, primarily due to its capacity to engage the body’s immune system to combat tumor cells through activation or enhancemen (34).

Immunotherapy can be delineated into two distinct modalities. Monotherapy acts to impede the interaction between tumor cells and the immune system, thereby triggering the immune system’s assault on tumor cells. In contrast, employing a combined approach involving chemotherapy and immunotherapy serves to ameliorate the side effects stemming from chemotherapy (35). Ginsenoside is a natural steroid that enhances and regulates the immune function, which not only inhibits the proliferation of lung adenocarcinoma, induces apoptosis of cancer cells, inhibits the inflammatory response of the body, but also plays a role in lung protection (36). Resveratrol is a natural polyphenolic compound with anti-free radical and antioxidant properties, which exerts anti-lung adenocarcinoma effects by modulating anti-inflammatory and hormonal effects (37). Therefore, the advantage of immunotherapy is that it allows tumors to achieve sustained remission and the adverse effects produced are relatively mild; as a popular treatment modality, immunotherapy, especially combination therapy, is widely used in the treatment of patients with lung adenocarcinoma, which creates more possibilities for the improvement of patients’ quality of survival.

### 2.6 Microbial gut flora therapy

The gut microbiota plays a pivotal role as an immunomodulator, exerting influence over the therapeutic response and effectiveness of certain immunotherapeutic agents. It has been established that the composition of the gut microbiota can correlate with both positive and negative treatment outcomes (38). Consequently, research into the tumor gut microbiome has been a topic of fervent discussion in recent years.

Several studies have illuminated the potential of interventions like Bifidus tetra punctate tablets to enhance the gut microbiota of lung adenocarcinoma patients. These interventions have shown significant promise, not only in regulating the patient’s intestinal microecology and augmenting immunity but also as adjunctive antitumor therapies when combined with conventional chemotherapy, with minimal drug toxicity (39). Subsequent research has revealed notable disparities in the gut flora of lung cancer patients compared to individuals with benign lung lesions, further substantiating the existence of specific intestinal microorganisms within the tumor population (40). These findings not only underscore the significance of the gut microbiota in lung adenocarcinoma but also open up new avenues for its treatment.

### 2.7 Summary

In recent years, Western medical approaches to lung adenocarcinoma have indeed achieved significant breakthroughs. This progress encompasses not only a diverse array of treatment modalities but also notable enhancements in the overall survival rates of patients. Nevertheless, it is essential not to overlook the associated challenges. Western medical treatment may give rise to issues such as toxic side effects, adverse reactions, drug resistance, and immune evasion. These phenomena consistently impact the quality of life of patients, and addressing these challenges represents a crucial imperative for the medical community moving forward. The advantages and disadvantages of Western medicine in the treatment of lung adenocarcinoma are shown in Table 1.

**Table 1:** Advantages and disadvantages of western medicine in the treatment of lung adenocarcinoma.

Treatment mode	Categorization	Methodologies	Advantages	Disadvantages
Western medicine treatments	Surgical interventions	Resection	Radical resection	Wound pain, shortness of breath, fatigue and loss of appetite
	Cytotoxic chemotherapy	Platinum, paclitaxel and other chemotherapeutic agents	Improves survival and delays metastasis to other organs	Adverse reactions such as malignant vomiting and myelosuppression
	Targeted pharmacotherapy	Specific molecules: EGFR, ALK, KRAS, ROS1, and BRAF, etc.	Inhibition of lung cancer cell growth, antitumor activity and enhancement of survival rate	Drug resistance, immune escape and toxic side effects
	Radiotherapy	High-energy rays	Improvement of survival rate Localized consolidation interventions	Fatigue, loss of appetite and mood swings
	Immunotherapy	Activation and enhancement of the immune system	Improvement of survival rates	Toxic side effect
	Microbial gut flora therapy	Regulates intestinal microecology and improves immunity	Lower drug toxicity	Fatigue and loss of appetite

## 3 Status of traditional Chinese medicine treatment of lung adenocarcinoma

The most significant departure from Western medicine lies in its emphasis on holistic regulation. Chinese medicine perceives a close interconnection between tumor development and the status of other internal organs such as the spleen, stomach, liver, and kidneys (16). Consequently, traditional Chinese medicine aims to harmonize these organs, fostering the revitalization of vital qi, with the overarching goal of bolstering the body’s resilience, combating cancer, and dispersing nodules through a comprehensive and synergistic approach. The principle of “supporting the positive and dispelling the evil” constitutes a fundamental tenet in the treatment of malignant tumors, aiming to enhance the patient’s overall immunity (41). This approach aligns closely with the core concept of immunotherapy in Western medicine, which seeks to mitigate drug resistance and prevent tumor immunity evasion. In both traditional Chinese medicine and Western medicine, the shared objective is to eliminate cancer cells within the body by stimulating the regrowth of immune cells and enhancing immune function. Additionally, these therapies work toward regulating the tumor microenvironment, thereby contributing to the treatment of lung adenocarcinoma. In the context of traditional Chinese medicine, lung adenocarcinoma is categorized as a syndrome associated with “plaque” and “lung accumulation.” It is often attributed to deficiencies in positive qi and insufficient spleen qi. Treatment approaches typically revolve around nurturing the earth element and generating gold, fortifying the Middle Earth, and bolstering qi. One notable example is the “soup from the Discussion of Internal and External Injuries,” which epitomizes this methodology (42). Given the intricate and varied disease presentations among lung adenocarcinoma patients, traditional Chinese medicine adopts a dialectical treatment approach that underscores the importance of “when the positive qi is in memory, the evil can not be interfered with.” This involves focusing on the sonification of the spleen and kidney, strengthening and safeguarding the positive qi while concurrently addressing detoxification and the dispersal of nodules. The treatment strategy further encompasses activating blood circulation, dispelling blood stasis to counteract the toxicity of cancer, and is marked by an individualized and flexible use of herbal medicines (43).

### 3.1 Herbal medicine for the treatment of lung adenocarcinoma

Traditional Chinese medicine (TCM) often categorizes lung adenocarcinoma into four distinct types based on the disease’s different stages and characteristics: deficiency of lung spleen and qi, deficiency of qi and yin, internal obstruction of blood stasis and toxicity, dampness and phlegm obstruction. Treatment is then tailored to these specific types and stages, with a primary focus on supporting the positive qi as the initial step, followed by attacking the pathogenic factors and ultimately complementing treatment with tonification (44). The primary objective of traditional Chinese medicine in the treatment of lung adenocarcinoma is to regulate the body’s immune function, induce apoptosis in tumor cells, and inhibit tumor cell proliferation. There are many compounds with medicinal value in flavonoids, which not only have cough suppressant, expectorant, asthma, and antibacterial activities but also have anti-free radical and antioxidant effects. In clinical flavonoids practice, certain herbs like Lobelia and Mulberry leaf have been noted for their ability to clear heat, remove toxins, reduce swelling, and alleviate pain, making them valuable in the treatment of lung cancer (45). Scutellaria barbata, for example, is employed for dispersing blood stasis, halting bleeding, promoting diuresis, and reducing swelling, making it suitable for addressing conditions like lung carbuncle, pulmonary tuberculosis with hemoptysis, and various types of bruises and injuries (46).

Recent reports have also highlighted the anticancer efficacy of active ingredients found in herbs that promote blood circulation and alleviate blood stasis. These components significantly enhance hemodynamics and the microenvironment, reduce thrombosis, boost blood flow, and subsequently inhibit lung cancer invasion and metastasis (47). The advantage of traditional Chinese medicine in treating lung adenocarcinoma is that it can provide evidence-based treatment according to the cause and development of the patient’s disease, enhance the immunity of the patient’s body, improve the quality of life, and prolong the growth period.

### 3.2 Acupuncture for lung adenocarcinoma

Acupuncture and moxibustion represent traditional Chinese medicine therapies rooted in the principles of yin and yang, the five elements, qi, blood, bodily fluids, internal organs, meridians, and the dynamics of the five movements and six qi (48). In the context of treating lung adenocarcinoma, acupuncture, and moxibustion primarily aim to enhance the circulation of qi and blood, promote the smooth flow of qi, and mobilize qi. It’s crucial to note that while acupuncture and moxibustion can provide valuable support, they cannot cure lung adenocarcinoma and should be considered as complementary therapies.

The selection of specific acupuncture points and needling techniques is tailored to the individual patient’s condition, with the objective of either tonifying deficiencies or promoting detoxification through various approaches. If the patient exhibits weakness in positive qi, tonification methods are employed; conversely, if pathogenic factors are dominant, detoxification methods are applied, or a combination of both strategies may be utilized. Ultimately, the goal is to strengthen the positive qi and dispel pathogenic influences (49).

Studies have demonstrated the benefits of acupuncture in alleviating lung cancer-related symptoms such as cough and phlegm, facilitating phlegm expectoration, and reducing the frequency of coughing (50). When used in conjunction with pharmacological interventions, it can yield significant analgesic effects (51). Acupuncture treatment has shown promising benefits in reducing patients’ adverse effects, relieving pain, and controlling tumor persistence.

### 3.3 Acupressure therapy for lung adenocarcinoma

Tui na practitioners employ a variety of techniques, including massage, manual acupoint stimulation, and structural adjustments, to rebalance the body’s state based on the specific needs of their patients. In the context of treating lung adenocarcinoma, tui na serves to promote blood circulation and alleviate pain (52). The primary techniques utilized in tui na include kneading, pinching, pushing, holding, and pressing, with a focus on areas related to the lungs and the back.

Research has indicated that daily kneading and pressing along the lung meridian represents an effective tui na method that can stimulate the functioning of internal organs, thus contributing to the equilibrium of yin and yang within the body (53). Appropriate massage techniques help facilitate phlegm discharge, enhance blood circulation, and alleviate symptoms like chest tightness, thereby ensuring the patient’s respiratory tract remains clear and relieving lung cancer-related symptoms such as cough, chest tightness, and shoulder and back pain (54). The most significant advantage of Tui Na treatment for lung adenocarcinoma is that it relieves the patient’s pain and can also be used as an adjunct to acupuncture as a way to increase the range of motion in the joints.

### 3.4 Summary

Chinese medicine treatment of lung adenocarcinoma exhibits several distinctive characteristics, including a multifaceted approach, consideration of multiple perspectives, and targeting various facets of the condition. Currently, Chinese medicine treatment for lung adenocarcinoma has evolved and is demonstrating promising results, whether used independently or in conjunction with Western medicine. However, it’s essential to acknowledge that traditional Chinese medicine’s effectiveness in treating lung adenocarcinoma is not universally precise. While these therapies primarily focus on safeguarding lung function, they may not consistently inhibit cancer cell growth or exhibit potent cytotoxic effects on lung cancer cells. Furthermore, there remains a lack of standardized diagnostic and treatment criteria within traditional Chinese medicine for lung adenocarcinoma. The absence of unified typing standards and evaluation criteria contributes to the complexity of treatment. Consequently, relying solely on Chinese medicine for the treatment of lung adenocarcinoma may not guarantee a complete cure. Instead, it is often advisable to incorporate Chinese medicine as a complementary therapy alongside Western medical approaches to optimize the chances of success and enhance the overall therapeutic outcome. The advantages and disadvantages of Chinese medicine in the treatment of lung adenocarcinoma are shown in Table 2.

**Table 2:** Advantages and disadvantages of Chinese medicine in treating lung adenocarcinoma.

Treatment mode	Categorization	Methodologies	Advantages	Disadvantages
Chinese medicine treatments	Chinese medicine	Regulates the immune function of the body	Clear heat, remove toxins, reduce swelling and alleviate pain, improving hemoptysis and microenvironment	As an adjunctive therapy only
	Acupuncture	Enhance the circulation of qi and blood, promote the smooth flow of qi, and mobilize qi	Analgesic	As an adjunctive therapy only
	Acupressure therapy	Regulates the body’s homeostasis	Promotes blood circulation and relieves chest tightness	As an adjunctive therapy only

## 4 Treatment of lung adenocarcinoma with the combination of traditional Chinese and Western medicine

### 4.1 Combined operation of traditional Chinese medicine

Surgical treatment is often considered the best approach for patients in the early stages of lung adenocarcinoma to achieve a radical cure. However, some patients may experience various uncomfortable symptoms post-surgery, including mood disorders, wound pain, asthma, fatigue, and loss of appetite. These symptoms can significantly impact the patient’s quality of life and hinder their postoperative recovery (55). Some patients are also intolerant to surgical treatment, and there are also problems such as large postoperative trauma, susceptibility to complications, and recurrence rates. Western medical treatments may have limitations in addressing these specific aspects of postoperative discomfort. Chinese medicine, rooted in a “people-oriented” treatment philosophy, offers a holistic approach that can provide valuable support for patients during their recovery (56). Research has indicated that the use of flavonoids in Chinese medicine as an adjuvant therapy following radical surgery can have several benefits for non-small cell lung cancer (NSCLC) patients. This approach has been shown to extend the survival period and enhance the quality of life for these patients. Additionally, Chinese medicine adjuvant therapy may reduce the rate of tumor recurrence and metastasis in lung cancer patients (57). Studies conducted by Li TM and others, employing Chinese herbal medicine (CHM) from Taiwan as a postoperative treatment for lung cancer patients, demonstrated a significant improvement in patient survival. The results indicated a lower mortality risk ratio in the group receiving CHM, highlighting the potential of Chinese medicine as an adjuvant therapy in reducing mortality rates among lung cancer patients (58).

### 4.2 Chinese medicine combined with Western medicine chemical treatment

For patients with intermediate and advanced lung adenocarcinoma, conventional treatment typically revolves around chemotherapy, with a common regimen involving platinum-containing double drugs. This chemotherapy approach is versatile and can be applied as a standalone treatment or in combination with other modalities, allowing for tailored treatment plans based on the patient’s specific condition. These plans may encompass preoperative treatment, postoperative adjuvant therapy, or late-stage treatment. However, chemotherapy, especially when used in combination, can come with side effects that some patients may find challenging to tolerate. Common adverse reactions include malignant vomiting and myelosuppression, which can lead to a reduced capacity for chemotherapy (59). For neoadjuvant chemotherapy, although it can control the growth of tumor cells, the disadvantages of adverse effects and reduced patient tolerance to surgery should not be ignored. Traditional Chinese medicine, grounded in the principle of maintaining the dynamic balance of the body, offers an alternative approach.

Song et al. (60) found that triptolide, as a world-recognized natural antitumor drug, can specifically target different cell signaling pathways associated with lung cancer progression; when co-administered with cisplatin, it can inhibit the expression of the nuclear factor κB (NFκB) signaling pathway and NFκB-regulated drug-resistance genes, and thus play a role in drug-resistant retroviral processes. Research conducted by Guo et al. (61) divided patients with stage IV lung adenocarcinoma into two groups: one receiving platinum-based chemotherapy alone and the other receiving a combination of traditional Chinese medicine alongside chemotherapy. The results indicated a noteworthy difference in survival outcomes. Patients who received only chemotherapy had a one-year survival rate of just 27%, which fell short of the average survival period (with an average survival time of 5 months). In contrast, patients who received traditional Chinese medicine alongside chemotherapy achieved a one-year survival rate of 88%, with an average survival time of 27 months. This difference was statistically significant (*p* < 0.0001), highlighting the potential of traditional Chinese medicine to enhance the survival rate of stage IV lung adenocarcinoma patients undergoing second-line chemotherapy.

In a study conducted by Lu et al. (62), 60 patients with advanced lung adenocarcinoma were randomly divided into two groups: the control group consisting of 27 patients, and the observation group consisting of 33 patients. The control group received pemetrexed combined with cisplatin regimen chemotherapy, while the observation group received Shengmai capsules in addition to the control group’s treatment. The study’s results revealed that the effective rate was 60.61% in the observation group and 62.96% in the control group. Importantly, the difference between the two groups was not statistically significant (*p*>0.05). Additionally, when comparing serum carcinoembryonic antigen (CEA) levels between the two groups before treatment (22.36 + 5.35 μg/L in the control group, 21.19 + 4.69 μg/L in the observation group), there was no statistically significant difference (*p*>0.05). However, after treatment, both groups exhibited lower CEA levels compared to before treatment (18.77 + 5.92 μg/L in the control group, 13.57 + 5.59 μg/L in the observation group), with the CEA level in the observation group being lower than that in the control group (*p*<0.05). This outcome suggests that the combination of traditional Chinese medicine with a chemotherapy regimen can mitigate the toxic effects of chemotherapy.

In light of these findings, it is evident that tailoring treatment approaches to individual patient differences in clinical practice, such as combining chemotherapy with traditional Chinese medicine treatment, can effectively reduce toxicity while improving patients’ survival prognosis and overall clinical outcomes.

### 4.3 Chinese medicine combined with targeted drug therapy

Targeted drug therapy is primarily designed to inhibit the proliferation of tumor cells and induce apoptosis by acting on specific targets that play a role in tumor cell growth and metastasis. While targeted drug therapy has revolutionized personalized cancer treatment in modern oncology, it also comes with challenges such as drug resistance, immune evasion, and various toxic side effects, including skin rashes, diarrhea, and liver function impairment (63). These adverse reactions can be severe enough to cause some patients to discontinue treatment, ultimately limiting the clinical utility of targeted drugs and the potential societal benefits they offer. In traditional Chinese medicine, the adverse reactions associated with targeted drug therapy are often attributed to pathogenic factors such as heat and toxicity. For these types of adverse reactions, the treatment approach typically involves clearing heat, detoxifying, and dispersing wind to expel the pathogenic factors from the body.

Jiao et al. (64) conducted a study to investigate the combination of traditional Chinese and Western medicine in lung adenocarcinoma patients to delay acquired resistance to epidermal growth factor receptor tyrosine kinase inhibitor (EGFR-TKI). Patients were randomly assigned to two groups: the experimental group received EGFR-TKI along with traditional Chinese medicine, while the control group received EGFR-TKI alone. The results revealed that the survival period of the EGFR-TKI + traditional Chinese medicine group was 13.50 months, whereas the EGFR-TKI group had a survival period of 10.94 months. This difference was statistically significant (*p* < 0.05), indicating a significant extension in overall survival for the experimental group. Additionally, the experimental group exhibited a significantly higher overall quality of life improvement rate (20.54%) compared to the control group (15.98%). This difference was statistically significant (*p* = 0.0160). These findings demonstrate that the combined treatment of traditional Chinese medicine with targeted drugs can effectively delay resistance to EGFR-TKI, enhance the quality of life for patients, and broaden the application of targeted drugs.

### 4.4 TCM combined with radiation therapy

Radiotherapy is a crucial approach in cancer treatment, utilizing radiation to target and treat tumor lesions. It is known for its localized application and wide range of therapeutic applications, offering targeted treatment options (22). However, the side effects of radiotherapy should not be ignored. After radiotherapy, patients with mild symptoms will experience fatigue, loss of appetite, and emotional uncertainty; some patients who have received radiotherapy for a long period will experience skin damage, esophageal damage, and lung damage, and in severe cases, they will experience radiation pneumonitis, radiation esophagitis, and bone marrow suppression, etc. (65). Incorporating Chinese medicine as an adjunct to radiotherapy can effectively mitigate the toxic side effects experienced by patients following radiotherapy. It helps alleviate post-radiotherapy discomfort, enhances patient compliance, and improves treatment efficacy (66).

In a study led by Du et al. (67), gefitinib and three-dimensional conformal radiotherapy were employed for treating 60 patients with advanced lung adenocarcinoma presenting with symptoms of breath and yin deficiency. Additionally, traditional Chinese medicine was integrated into the treatment protocol by administering Guiqi Yiyuan Cream orally to another 60 patients in the control group. The study outcomes revealed significant differences in favor of the treatment group. After 7 weeks of continuous treatment, the treatment group exhibited a higher total effective rate (70.00% vs. 51.57%) and total control rate (86.67% vs. 80.00%) compared to the control group, with these differences being statistically significant (*p* < 0.05). Moreover, the incidence rates of malignancy, diarrhea, and rash in the control group were notably higher (65.00, 20.00, and 13.33%, respectively) than those in the treatment group (28.33, 6.67, and 3.33%, respectively), and these differences were statistically significant (*p* < 0.05 or *p* < 0.01). These findings underscore the remarkable clinical efficacy of combining Guiqi Yiyuan Cream with gefitinib and radiotherapy in treating advanced lung adenocarcinoma with breath and yin deficiency syndrome. This combined approach significantly improves patient prognosis and extends their survival. Traditional Chinese medicine offers unique advantages in reducing the adverse effects of radiotherapy while enhancing tumor tissue sensitivity to radiation and thereby improving treatment effectiveness. Some studies have demonstrated that traditional Chinese medicine’s heat-clearing and detoxification properties, as well as its ability to promote blood circulation and eliminate blood stasis, can sensitize tumor tissues to radiotherapy (68).

### 4.5 TCM combined with immunotherapy

The introduction of immune checkpoint inhibitors marked a significant advancement in the treatment of early-stage tumors, offering the potential for personalized cancer treatment by harnessing the body’s natural tumor-fighting mechanisms (69). While targeted therapies like immunotherapy have shown great promise in treating cancer, they also come with the challenge of identifying and managing their toxic side effects. These side effects can include conditions like rash, itching, diarrhea, thyroid dysfunction, colitis, and pneumonia. Western medicine approaches immunotherapy with careful consideration of each patient’s variability and sensitivity to these toxicities. Tailored dosing is often necessary to optimize the benefits while minimizing adverse effects (70).

Here, traditional Chinese medicine can play a valuable role as an adjuvant therapy following immunotherapy. For instance, Qingzao Lung Rescue Soup has been found to effectively extend the survival period of non-small cell lung cancer patients, enhance the host’s immune response, and improve overall survival rates (71). Acupuncture, too, offers advantages in immunotherapy for non-small cell lung cancer. Scholars like Mao Jinfeng and colleagues have discovered that wheat grain moxibustion at the foot-sanli point can complement Western medicine treatments for advanced non-small cell lung cancer, boosting the immune function of patients who are tumor-free post-surgery. This combination treatment approach is well-received, well-tolerated, and safe, making it a promising option for clinical use (72). The cases and efficacy of combined Chinese and Western medicine in the treatment of lung adenocarcinoma are shown in Table 3.

**Table 3:** Case and efficacy of combined Chinese and Western medicine in the treatment of lung adenocarcinoma.

Treatment mode	Categorization	Examples	Healing effect
Combine traditional Chinese and Western medicine	Combined operation of traditional Chinese medicine	Chinese herbal medicine care after surgical treatment	Reducing mortality
	Chinese medicine Combined with Western medicine chemical treatment	Shengmai capsule was given on the basis of pemetrexed combined with cisplatin	Reduce chemotherapy toxicity Improve survival prognosis
	Chinese medicine combined with targeted drug therapy	Targeting EGFR-TKI drugs in combination with traditional Chinese medicine	Delaying EGFR-TKI resistance Improving patients’ quality of life Expanding the scope of targeted drugs
	TCM combined with radiation therapy	Combination of Gefitinib and three-dimensional conformal radiotherapy with Guiqi Yiyuan Cream	Significantly improves patient prognosis Reduces toxicity and increases sensitivity Prolongs patient survival
	TCM combined with immunotherapy	Immune checkpoint inhibitor combined with Qingzao Lung Rescue Soup	Enhances the host’s immune response Improves the overall survival of patients

## 5 Summary

Over the years, a range of treatment modalities, including surgery, chemotherapy, targeted drugs, radiation therapy, immunotherapy, and interventions in the intestinal flora, have played pivotal roles in managing lung adenocarcinoma across its different stages. These treatments are continually evolving with advancements in society and medical science. However, it’s important to acknowledge that these Western treatments often come with varying degrees of adverse effects for patients. Chinese medicine, with its emphasis on the holistic balance of the body and a “patient-centric” treatment approach, presents a significant advantage when combined with Western medicine. The integration of Chinese medicine into Western medicine regimens helps mitigate the drawbacks of Western treatments (73). This combined approach can contribute to improved patient well-being by reducing toxic side effects, preventing tumor recurrence and metastasis, stabilizing the patient’s emotional state, alleviating clinical symptoms, and extending the patient’s survival period.

The effectiveness of combined Chinese and Western medicine treatment compared to individual medication still lacks robust multi-center, randomized controlled, large-sample clinical data for confirmation. Additionally, the field of Chinese medicine lacks standardized criteria for the dialectical classification of lung adenocarcinoma and evaluation criteria for the disease. Further, there is limited experimental validation of the interactions between Chinese and Western medicine treatments for lung adenocarcinoma and how these interactions affect the internal environment of the body. Research into the mechanisms underlying the reduction of toxicity in combined Chinese and Western medicine treatment is also a promising future direction that requires rigorous investigation through high-quality clinical studies.

**Figure 1:**
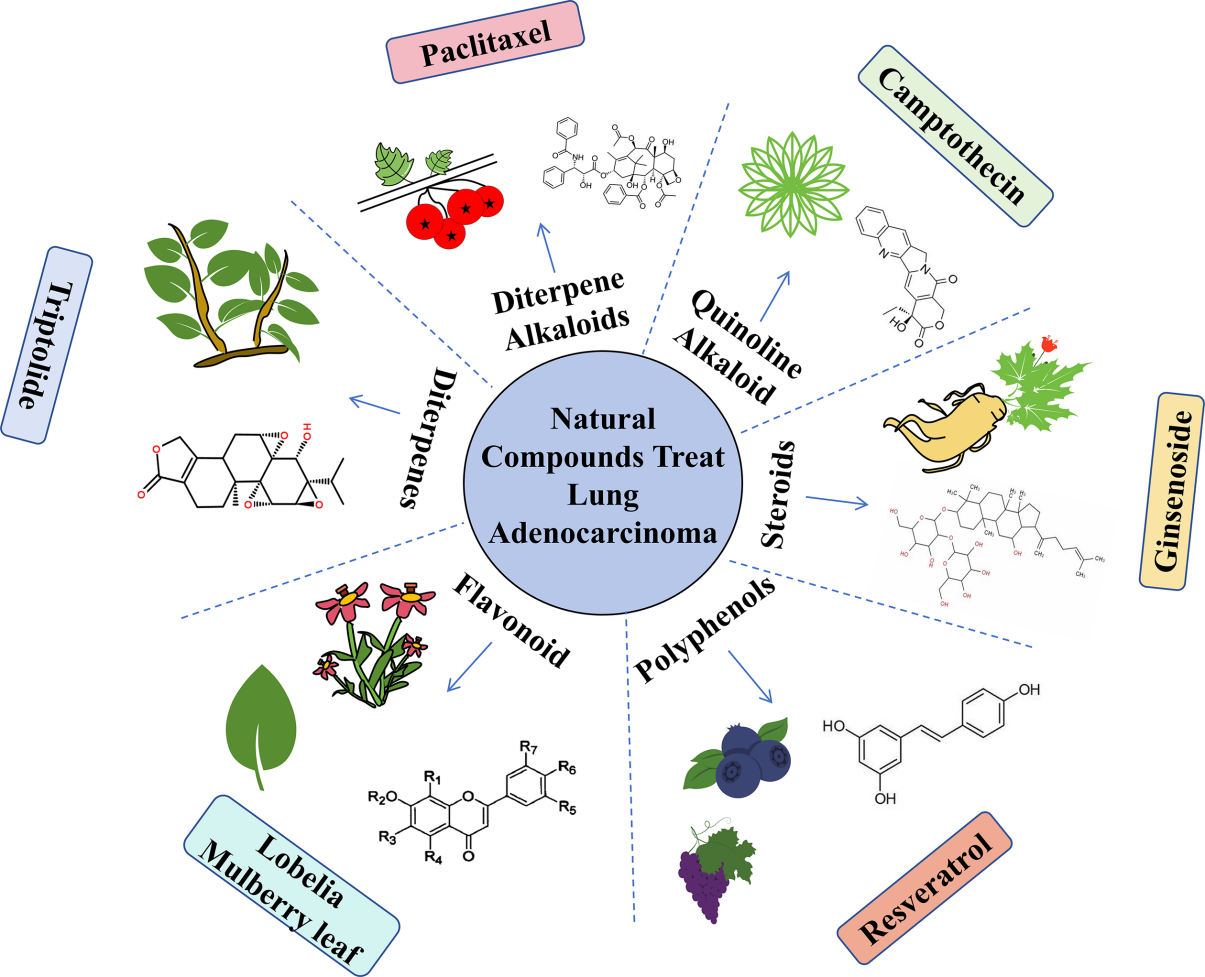
Natural compounds used to treat lung adenocarcinoma.

**Figure 2:**
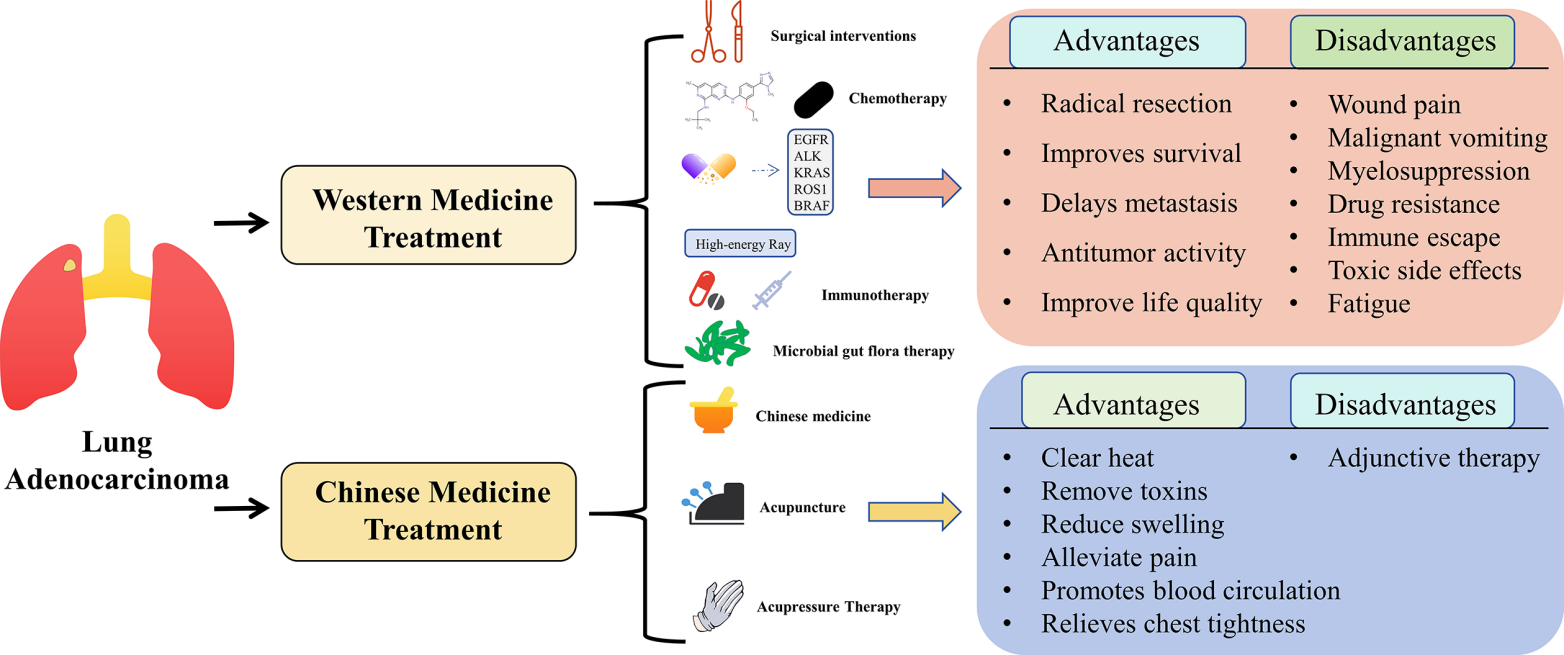
Western medicine compared with Chinese medicine in treating lung adenocarcinoma.

In conclusion, the integration of Chinese and Western medicines in cancer treatment remains a major area of interest and will continue to advance based on a solid and innovative theoretical foundation. We anticipate breakthroughs in this field that will bring hope to patients with cancer. Natural compounds mentioned in this article are shown in Figure 1. The comparison between Western medicine and Chinese medicine in the treatment of lung adenocarcinoma is shown in Figure 2.

## Author contributions

HJ: Conceptualization, Data curation, Formal analysis, Investigation, Writing – original draft. LB: Project administration, Supervision, Writing – review & editing.
